# Performance of mutation pathogenicity prediction tools on missense variants associated with 46,XY differences of sex development

**DOI:** 10.6061/clinics/2021/e2052

**Published:** 2021-01-18

**Authors:** Luciana R. Montenegro, Antônio M. Lerário, Miriam Y. Nishi, Alexander A.L. Jorge, Berenice B. Mendonca

**Affiliations:** I Unidade de Endocrinologia do Desenvolvimento / LIM42 / SELA, Disciplina de Endocrinologia, Hospital das Clinicas (HCFMUSP), Faculdade de Medicina, Universidade de Sao Paulo, Sao Paulo, SP, BR; II Division of Metabolism, Department of Internal Medicine, Endocrinology and Diabetes, University of Michigan, Ann Arbor, United States of America; III Unidade de Endocrinologia Genetica (LIM25), Disciplina de Endocrinologia, Faculdade de Medicina (FMUSP), Universidade de Sao Paulo, Sao Paulo, SP, BR

**Keywords:** Disorders of Sex Development, Pathogenicity Prediction Tools, Genetics

## Abstract

**OBJECTIVES:**

Single nucleotide variants (SNVs) are the most common type of genetic variation among humans. High-throughput sequencing methods have recently characterized millions of SNVs in several thousand individuals from various populations, most of which are benign polymorphisms. Identifying rare disease-causing SNVs remains challenging, and often requires functional *in vitro* studies. Prioritizing the most likely pathogenic SNVs is of utmost importance, and several computational methods have been developed for this purpose. However, these methods are based on different assumptions, and often produce discordant results. The aim of the present study was to evaluate the performance of 11 widely used pathogenicity prediction tools, which are freely available for identifying known pathogenic SNVs: Fathmn, Mutation Assessor, Protein Analysis Through Evolutionary Relationships (Phanter), Sorting Intolerant From Tolerant (SIFT), Mutation Taster, Polymorphism Phenotyping v2 (Polyphen-2), Align Grantham Variation Grantham Deviation (Align-GVGD), CAAD, Provean, SNPs&GO, and MutPred.

**METHODS:**

We analyzed 40 functionally proven pathogenic SNVs in four different genes associated with differences in sex development (DSD): 17β-hydroxysteroid dehydrogenase 3 (HSD17B3), steroidogenic factor 1 (NR5A1), androgen receptor (AR), and luteinizing hormone/chorionic gonadotropin receptor (LHCGR). To evaluate the false discovery rate of each tool, we analyzed 36 frequent (MAF>0.01) benign SNVs found in the same four DSD genes. The quality of the predictions was analyzed using six parameters: accuracy, precision, negative predictive value (NPV), sensitivity, specificity, and Matthews correlation coefficient (MCC). Overall performance was assessed using a receiver operating characteristic (ROC) curve.

**RESULTS:**

Our study found that none of the tools were 100% precise in identifying pathogenic SNVs. The highest specificity, precision, and accuracy were observed for Mutation Assessor, MutPred, SNP, and GO. They also presented the best statistical results based on the ROC curve statistical analysis. Of the 11 tools evaluated, 6 (Mutation Assessor, Phanter, SIFT, Mutation Taster, Polyphen-2, and CAAD) exhibited sensitivity >0.90, but they exhibited lower specificity (0.42-0.67). Performance, based on MCC, ranged from poor (Fathmn=0.04) to reasonably good (MutPred=0.66).

**CONCLUSION:**

Computational algorithms are important tools for SNV analysis, but their correlation with functional studies not consistent. In the present analysis, the best performing tools (based on accuracy, precision, and specificity) were Mutation Assessor, MutPred, and SNPs&GO, which presented the best concordance with functional studies.

## INTRODUCTION

The term “differences in sex development” (DSD) refers to congenital conditions in which chromosomal, gonadal, or anatomical sex development is atypical (1). They can be classified into three major categories: sex chromosome DSDs, 46,XX DSDs, and 46,XY DSDs (2). Most causes of DSDs are genetically determined, and several genes have been found to be associated with the DSD phenotype (3). Recent studies in individuals with DSDs have characterized numerous single nucleotide variants (SNV) in several genes, most of which are benign polymorphisms. However, distinguishing rare disease-causing SNVs from rare polymorphisms remains challenging. Functional studies for disease association variants are often used, but are laborious and time-consuming (4,5).

Many methods have been developed for the computational prediction of the pathogenicity of SNVs, which are based on evolutionary conservation, protein structure/function, or assembly parameters, such as allelic diversity, pathogenicity, and association with genome-wide association studies (6). Studies analyzing the performance of prediction programs have been completed using a large number of missense variants (7). In the present study, we compared the performance of 11 widely used pathogenic prediction tools in the analysis of proven pathogenic DSD-causing SNVs in four different genes.

## MATERIAL AND METHODS

### Dataset

We analyzed 40 disease-causing SNVs in four different genes associated with DSD: 17β-hydroxysteroid dehydrogenase 3 (HSD17B3), steroidogenic factor 1 (NR5A1), androgen receptor (AR), and luteinizing hormone/chorionic gonadotropin receptor (LHCGR). All pathogenic allelic variants have been published with functional studies showing loss of function activity (Table 1). To evaluate the false discovery rate of each tool, we selected 36 frequent benign SNVs (MAF>0.01) found in the same DSD genes (Table 1).

### Prediction Methods

We selected 11 widely used pathogenic prediction tools freely available on the Web: Fathmn, Mutation Assessor, Protein Analysis Through Evolutionary Relationships (Phanter), SIFT (Sorting Intolerant From Tolerant), Mutation Taster, Polymorphism Phenotyping v2 (Polyphen-2), Align Grantham Variation Grantham Deviation (Align-GVGD), CAAD, Provean, and SNPs&GO (Table 2).

### Statistical Analysis

The quality of the predictions was analyzed using six parameters: accuracy, precision, negative predictive value (NPV), sensitivity, specificity, and Matthews correlation coefficient (MCC). In the equations below, tp, tn, fp, and fn refer to true positive, true negative, false positive, and false negative, respectively.[Fig f01]

The MCC (43) is an important statistics tool that is widely used in bioinformatics as a performance metric, as it is not affected by the differing proportions of neutral and pathogenic datasets predicted by the different programs. Additionally, we also assessed the overall performance of deleterious prediction with the receiver operating characteristic (ROC) curve and area under the curve (AUC), using MedCalc for Windows, version 15.0 (MedCalc Software, Ostend, Belgium). ROC curves are an indicator of probability and performance for classification problems at various threshold settings, and AUCs represent the degree or measure of separability. Together, they indicate how capable a model is of distinguishing between classes. The higher the AUC, the better the model is at predicting an outcome (44).

We randomized the results of prediction of SNV present in the DDS genes as pathogenic or benign, based on the classification given by each program. The classifications “probably benign”, “benign”, “low”, and “neutral”, provided by Fathmn, Mutation Assessor, Phanter, SIFT, Mutation Taster, Provean, and Polyphen-2, were considered as benign. The classifications “possibly damaging”, “probably damaging”, “high”, “medium”, “deleterious”, and “damaging”, were considered as pathogenic. For CAAD, which uses numeric scores, values ≤10 were classified as benign, and those >10 were classified as pathogenic. Align-GVGD also used numeric scores, which were classified as benign up to 25, and anything above was classified as pathogenic.

## RESULTS

Based on the results for each program, none of the tools were 100% precise in identifying pathogenic SNVs. The values for the parameters measured are listed in Table 3, and include all pathogenic and benign variants. Phanter had the highest precision in the classification of pathogenic variants (38 out of 40 known to be pathogenic), followed by Mutation Taster and Polyphen-2 (both 37 out of 40 known to be pathogenic). Align-GVGD correctly classified fewer known pathogenic SNVs than any other tool (33 of 40 known to be pathogenic). Phanter and Mutation Taster both classified a high number of know benign SNVs as pathogenic (21 and 17, respectively, of 36).

Mutation Assessor, MutPred, and SNPs&GO presented more consistent results regarding the nature of the SNVs (pathogenic or benign). MutPred had the highest accuracy, precision, and specificity (0.83, 0.85, and 0.83, respectively), as seen in Table 4. Mutation Assessor has the highest sensitivity of all the tools evaluated, although five other tools (Phanter, SIFT, Mutation Taster, Polyphen-2, and CAAD) exhibited sensitivity >0.90, however, they were found to have lower specificity (0.42-0.67). Based on MCC, performance ranged from poor (Fathmn=0.04) to reasonably good (MutPred=0.66). Fathmn and Align-GVGD exhibited the worst performance, with a high number of false positive results (MMC=0.04 and 0.06, respectively).

**Figure 1:**
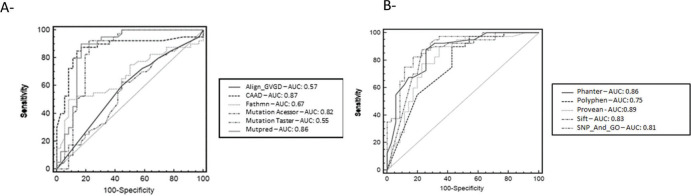
ROC curves of 11 methods separated into random groups: **A -** Align-GVD, CAAD, Fathmn, Mutation Assessor, Mutation Taster and MutPred; **B -** Phanter, Polyphen-2, Provean, SIFT, and SNPs&GO.

The comparative predictive performance of each tool was evaluated using the AUC scores from ROC plots and the true negative rate (TNR, or specificity) as measurements. The analysis was separated into random groups, since the program analyzed a maximum of six samples at a time (Figure 1). The AUC values varied from low (Mutation Taster=0.55) to reasonably good (SNPs-&-GO=0.89), and two programs (Fathmn and Align-GVD) for which the MMC values were poor (0.04 and 0.06, respectively), improved in the statistical analysis made using the ROC curve (0.67 and 0.57, respectively). The other programs did not see a change in their statistical values at the same level.

## DISCUSSION

In the present study, we analyzed and compared the abilities of 11 widely available tools for predicting the pathogenicity of SNVs. Although some algorithms are based on the same data sets, they differ in the database for conservation analysis and structural attributes. They also differ in the information required to run the predictions, as some programs request the accession number of the gene, others the protein change, nucleotide change, or chromosomal position.

Overall, we found that Mutation Assessor, MutPred, and SNPs&GO were the most reliable predictors for SNV classifications. They also exhibited the best AUC results. The accuracy of all tools evaluated ranged from poor to reasonably good (MMC=0.04-0.66). These results are consistent with what has been shown in previous studies (7,9), which is that the number of samples used in the analysis did not influence the statistical result as much.

In conclusion, computational algorithms are important screening tools for prioritizing and identifying disease-causing SNVs, but their correlation with functional studies is not consistent. In the present analysis, the highest-performing tools were Mutation Assessor, MutPred, and SNPs&GO.

## AUTHOR CONTRIBUTIONS

Montenegro LR contributed to the acquisition, analysis, interpretation of data, and drafting of the article. Lerario AM contributed to the interpretation of data and revising the article. Nishi MY contributed to the interpretation of data, drafting and revising the article. Jorge AA contributed to the analysis and interpretation of data. Mendonca BB contributed to the conception and design of the study, drafting and revising the article.

## Figures and Tables

**Figure f01:**
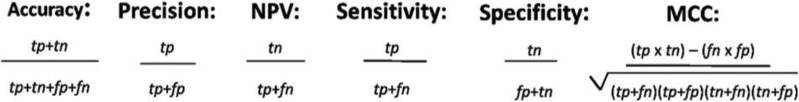


**Table 1:** Single Nucleotide Variants (SNV) in DSD-related genes used for prediction analysis.

Gene/Protein	Pathogenic allelic Variant	Benign allelic variant	Reference
HSD17B3 17β-hydroxysteroid dehydrogenase ENST00000375263 NP_000188	p.Ser65Leu	p.Val25Met	(8)
	p.Arg80Gln	p.Val31Leu	(8)
	p.Ala203Val	p.Gly289Arg	(9)
	p.Val205Glu	p.Ile102Phe	(8)
	p.Phe208Ile	p.Glu114Lys	(8)
	p.Glu215Asp	p.Arg45Trp	(8)
	p.Ser232Leu	p.Arg45Gln	(10)
	p.Met235Val	p.Ser65Ala	(10)
	p.Pro282Leu	p.Ile223Val	(8)
	p.Cys268Tyr		(11)
NR5A1 Steroidogenic factor 1 ENST00000373588.8 NP_004950	p.Val15Met	p.Glu11Asp	(11)
	p.Val20Leu	p.Gly146Ala	(12)
	p.His24Tyr	p.Val173Met	(12)
	p.Arg39Pro	p.Gly178Arg	(13)
	p.Met78Ile	p.Tyr211Cys	(11)
	p.Gly91Ser	p.Pro235Leu	(14)
	p.Pro235Leu	p.Thr296Met	(15)
	p.Trp279Arg	p.Val355Met	(13)
	p.Arg313Cys		(13)
	p.Leu437Gln		(14)
AR Androgen receptor ENST00000374690.8 NP_000035	p.Cys579Phe	p.Ala45Gly	(16)
	p.Phe582Tyr	p.Gln59Leu	(16)
	p.Arg710Thr	p.Gln87His	(17)
	p.Gly724Asp	p.Gln91Lys	(18)
	p.Gly750Asp	p.Gly216Arg	(17)
	p.Ala765Thr	p.Leu272Phe	(17)
	p.Arg774His	p.Leu341Met	(17)
	p.Leu812Pro	p.Val731Met	(18)
	p.Arg855Cys	p.Arg856Leu	(17)
	p.Asp864Gly		(17)
LHCGR Luteinizing hormone/chorionic gonadotropin receptor ENST00000294954 NP_000224	p.Ile374Thr*	p.Ala57Thr	(19)
	p.Thr392lle*	p.Ile103Lys	(19)
	p.Phe194Val*	p.Tyr113His	(20)
	p.Glu354Lys*	p.Ala118Glu	(21)
	p.Leu502Pro*	p.Lys126Asn	(22)
	p.Met398Thr**	p.Lys137Asn	(23)
	p.Leu547Arg**	p.Val144Leu	(24)
	p.Asp564Gly**	p.Phe611Val	(25)
	p.Ala568Val**	p.Cys543Tyr	(26)
	p.Ile575Leu**	p.Gly504Ser	(27)

* Inactivating variants - phenotype: Leydig cell hypoplasia.

** Activating variants - phenotype: GIPP (gonadotropin-independent precocious puberty).

**Table 2:** Basis of the *in silico* prediction algorithms.

Program Name	URL and Key reference	Basis	Reference
Fathmn	http://fathmm.biocompute.org.uk/	Evolutionary conservation	(28),(29),(30)
Mutation Assessor	http://mutationassessor.org/r3/	Evolutionary conservation	(31)
Phanter	http://www.pantherdb.org/	Evolutionary conservation	(32)
SIFT	https://sift.bii.a-star.edu.sg/	Evolutionary conservation	(33)
Mutation Taster	http://www.mutationtaster.org/	Protein structure/function and Evolutionary conservation	(34)
Polyphen-2	http://genetics.bwh.harvard.edu/pph2/	Protein structure/function and Evolutionary conservation	(35)
Align-GVGD	http://agvgd.hci.utah.edu/	Protein structure/function and Evolutionary conservation	(36)
MutPred	http://mutpred.mutdb.org/index.html	Protein structure/function and Evolutionary conservation	(37)
CAAD	https://cadd.gs.washington.edu/	Protein structure/function and Evolutionary conservation	(38)
Provean	http://provean.jcvi.org/index.php	Protein structure/function	(39)
SNPs&GO	http://snps.biofold.org/snps-and-go//snps-and-go.html	Protein structure/function	(40),(41),(42)

**Table 3:** Predictions by algorithms.

Type of SNV	Classification by the sites	Fathmn	Mutation Assessor	Phanter	SIFT	Mutation Taster	Polyphen-2	Align-GVGD	MutPred	CAAD	Provean	SNPs&GO
Pathogenic (n=40)	Pathogenic	35	38	38	36	37	37	33	33	36	32	32
	Benign	5	2	2	4	3	3	7	7	4	8	8
Benign (n=36)	Pathogenic	27	14	21	16	17	13	28	6	12	11	6
	Benign	9	22	15	18	19	13	8	30	24	25	30

**Table 4:** Performance of the prediction algorithms.

Performance	Fathmn	Mutation Assessor	Phanter	SIFT	Mutation Taster	Polyphen-2	Align-GVGD	MutPred	CAAD	Provean	SNPs&GO
Accuracy	0.56	0.79	0.70	0.74	0.74	0.76	0.54	0.83	0.79	0.75	0.82
Precision	0.56	0.73	0.64	0.69	0.69	0.73	0.54	0.85	0.75	0.74	0.84
Specificity	0.16	0.61	0.42	0.53	0.53	0.53	0.22	0.83	0.67	0.69	0.83
Sensitivity	0.88	0.95	0.95	0.92	0.93	0.93	0.83	0.83	0.90	0.80	0.80
NPV	0.50	0.92	0.88	0.86	0.86	0.84	0.53	0.81	0.86	0.76	0.79
MMC	0.04	0.60	0.44	0.50	0.50	0.51	0.06	0.66	0.59	0.50	0.63
